# Syndrome de Sweet gravidique: une entité rare à ne pas méconnaitre

**DOI:** 10.11604/pamj.2014.18.185.4881

**Published:** 2014-07-03

**Authors:** Wafa Chebbi, Olfa Berriche

**Affiliations:** 1 Service de Médecine Interne, CHU Taher Sfar Mahdia, 5100 Mahdia, Tunisie

**Keywords:** Plaques érythémato-papuleuses, syndrome de Sweet, membres inférieurs, grossesse, erythematous-papule plates, Sweet syndrome, lower limbs, pregnancy

## Image en médecine

Le syndrome de Sweet ou dermatose aiguë fébrile neutrophilique est une maladie inflammatoire rare à expression cutanée prédominante, appartenant au groupe des dermatoses neutrophiliques. Il est caractérisé par le polymorphisme de son expression clinique et la diversité des maladies qui peuvent lui être associées. Le syndrome de Sweet gravidique est rare (2%) et caractérisé par la récidive des lésions aux grossesses ultèrieures. Sa pathogénie est mal élucidée et serait probablement expliquée par un mécanisme hormonal. Nous rapportons l'observation d'une femme âgée de 24 ans, primigeste et enceinte de 25 semaines d'aménorrhée, qui consultait devant l'apparition brutale des lésions cutanées douloureuses des membres inférieurs, associées à une asthénie et des polyarthralgies inflammatoires des grosses articulations. L'examen physique trouvait une patiente fébrile à 38,5°C, une tachycardie à 96 battements par minute, des plaques érythémato-papuleuses, à bords nets, infiltrées, douloureuses à la palpation et siégeant au niveau des membres inférieurs. Il n'y avait pas d'arthrite ni de synovite. Le reste de l'examen était sans particularités. Le bilan biologique montrait une vitesse de sédimentation à 90 mm à la première heure, une protéine C-réactive à 20 mg/L et une hyperleucocytose à 13200 éléments/mm^3^ à prédominance polynucléaires neutrophiles. L’échographie abdominale et cardiaque était normale. Le bilan immunologique (AAN, ANCA, facteurs rhumatoïdes, anticorps anti-phospholipides) était négatif. La biopsie cutanée montrait un infiltrat dermique dense à polynucléaires neutrophiles sans vascularite leucocytoclasique, en faveur d'un syndrome de Sweet. Une corticothérapie à la dose de 0,5 mg/kg/j était instaurée. L’évolution était favorable avec disparition de la fièvre, des arthralgies et régression complète des lésions cutanées au bout d'une semaine.

**Figure 1:**
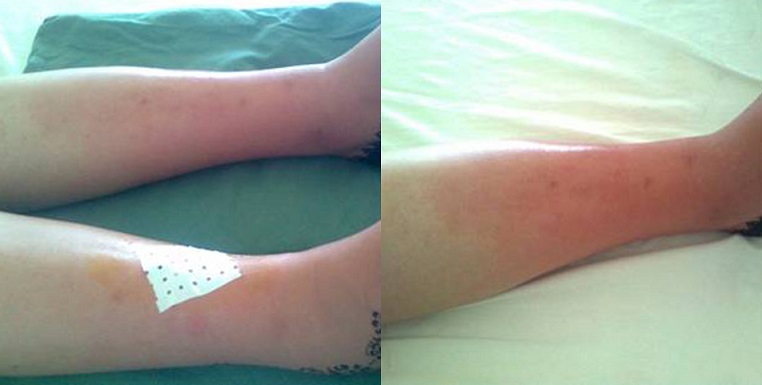
Plaques érythémato-papuleuses à bords nets des membres inférieurs

